# The Prevalence of Polypharmacy and the Contribution of Age, Period, and Cohort Effects in Sweden From 2006 to 2020

**DOI:** 10.1002/hsr2.70778

**Published:** 2025-05-19

**Authors:** Theodore T. Miao, Malin Ericsson, Jonas W. Wastesson

**Affiliations:** ^1^ Aging Research Center, Department of Neurobiology Care Sciences and Society, Karolinska Institutet & Stockholm University Stockholm Sweden; ^2^ Department of Medical Epidemiology and Biostatistics Karolinska Institutet Stockholm Sweden

**Keywords:** cohort analysis, drug, epidemiology, medication, polypharmacy

## Abstract

**Objective:**

To investigate the age, period, and cohort effects of polypharmacy in older adults using Swedish register data covering the period 2006 to 2020.

**Design:**

Repeated cross‐sectional study using routinely‐collected health care data.

**Setting:**

Nationwide, Sweden.

**Participants:**

A 10% random sample was drawn each year from the source population of all adults aged ≥ 65 in Sweden, 2006–2020 (cumulative *n* > 3,000,000).

**Measurement:**

Polypharmacy was defined as the use of ≥ 5 medicines. Drug data were extracted from the National Prescribed Drug Register (NPDR) Medication use was assessed on 1st January (1‐day point prevalence) each year based on the drug duration episodes. Age‐Period‐Cohort analysis was conducted to explore these effects.

**Results:**

Overall, 32.8% of older adults was exposed to polypharmacy in 2020 compared to 28.6% in 2006. This increase was more pronounced among individuals aged 85 to 89, from while the prevalence remained relatively steady among those aged 65 to 84. In the formal Age‐Period‐Cohort analysis, the cohort differences were weak for polypharmacy, but more prevalent for specific medication classes.

**Conclusion:**

Polypharmacy is mainly influenced by age and period effects, but not by cohort. The rise in polypharmacy is primarily propelled by an increased pace in medication use among individuals aged 75 to 89 years. These findings can provide valuable insights for making effective strategies aimed at reducing polypharmacy.

## Summary

- The prevalence of polypharmacy in the older adult population is increasing in Sweden, as elsewhere.
- The rising trend in the prevalence is related to a more substantial increase in the number of drugs used between the ages 75–89 rather than an increasing number when entering old age.
- Cohort effects had a weak role for the prevalence of polypharmacy but might be more pronounced for specific medications.

## 1 Introduction

Polypharmacy, the concurrent use of multiple drugs, is increasing in most populations of older adults. [1] Because polypharmacy has been associated with various negative clinical conditions, [1, 2, 3, 4] increasing attention has been given to understanding the factors driving polypharmacy. [5] Apart from identifying factors associated with polypharmacy, it is also essential to provide detailed evidence about key risk factors, such as age, and how these associations change over time.

The prevalence of polypharmacy in older adults has been on the rise since the 1980s [1, 6, 7, 8, 9] and is still increasing in Sweden. [9] Age period cohort (APC) analysis is useful for understanding time‐varying elements in epidemiology, For example to identify age groups or cohorts in the population with unfavorable development over time, so that interventions can be more precisely implemented. In relation to polypharmacy, higher age is consistently the most prominent risk factor of polypharmacy, [10] albeit the prevalence of polypharmacy tends to stagnate or decline after the age of 95 years. [11] Period effect represents the cross‐sectional environment a society experiences, [12] reflected by the increasing trend in polypharmacy. Last, cohort effects can be conceptualized as the interaction between age and period, “a period effect that is differentially experienced through age‐specific exposure or susceptibility to that event or cause”. [12, 13] In this specific study, cohort refers to individuals born within an specific interval of years. Cohort effects have been shown for specific medications, for example drugs with anticholinergic properties. [14] Especially since newly marketed drugs are often approved for a specific age category, specific birth cohorts are more likely than other to receive certain drugs. Cohort effects have rarely been discussed in relation to the prevalence of polypharmacy or drug use in general in older adults. [14] To date, there is limited knowledge about whether the increasing trend in polypharmacy have been shared by all age groups or cohorts of older adults.

A better understanding of how age, period, and cohort effects contribute to the secular increase in polypharmacy can help tailor interventions aimed at reducing the prevalence of polypharmacy. Thus, our aim is to investigate the age, period, and cohort effects of polypharmacy in older adults using Swedish register data covering the period 2006 to 2020. As a secondary aim, we repeated the analysis for the three most commonly prescribed drug categories separately.

## 2 Methods

### 2.1 Data Source

We used routinely collected administrative and health data with national coverage in Sweden from the Total Population Register, the National Prescribed Drug Register, and the Swedish Register of Education. Individuals were linked using pseudonymized identifiers.

### 2.2 Study Population and Study Design

A 10% random sample was drawn each year from the source population of all adults aged ≥ 65 in Sweden, 2006–2020 (*n* > 3,000,000). Sampling was done to reduce computer analysis time. This is a repeated cross‐sectional design covering the years 2006 to 2020.

### 2.3 Outcomes

Polypharmacy was classified as the concurrent use of five or more medications, which is the most frequently used definition. [15] Drug data were extracted from the National Prescribed Drug Register (NPDR) Medication use was assessed on 1st January each year based on the drug duration episodes according to Anatomical Therapeutic Chemical (ATC) codes level 5. [16] The total number of used drugs was calculated according to the same principle and included as an outcome in the sensitivity analyses.

The three most commonly used drug categories in the year 2020 were analyzed separately as a secondary outcome: agents acting on the renin‐angiotensin system (ATC code C09), antithrombotic agents (ATC code B01), and lipid‐modifying agents (ATC code C10). The selection of drugs that are most frequently prescribed is driven by their potential as the most effective targets for intervention aimed at deprescribing.

### 2.4 Covariates

Age was defined in 5‐year age categories from 65 to 99 years. All older than 99 years was in the last age group.

Period was divided in 1‐year categories.

Cohort was defined by birthyear and classified in 5‐year categories.

Education was defined according to International Standard Classification of Education (ISCED) 2011 [17] and categorized into “Pre‐secondary”, “High school”, and “University and higher”.

### 2.5 Statistical Analysis

We used descriptive statistics to describe the individual characteristics for the study population each year. The association between age, period, and cohort and the prevalence of polypharmacy as well as specific drug categories was tested by Age‐period‐cohort (APC) model and graphic analysis.

This identification problem of APC analysis, that is, that the variables are perfectly collinear are well known. [18] Several approaches to the identification problem have been suggested. We used the reparametrized classical APC model by Nielsen [18] to bypass the identification problem. The reparametrized classical APC model was introduced by Kuang, Nielsen and Nielsen, [19] and concluded into a framework by Fannon [20] to create a freely varying parameter using the second difference (acceleration) of time effects. The framework focuses on explaining the dependent variable with the nonlinear relationship with age, period, and cohort accelerations.

For example, for the age effect, the first difference is ∆α_i_, the second difference is ∆^2^α_i_ = ∆α_i_ − ∆α_i−1_. Thus, if the matrix is an age‐cohort coordinate system, it can be identified from the predictor via:

(1) $$\Delta^{2}\alpha_{i}=\mu_{\text{ik}}–\mu_{i-1,k+1}–(\mu_{i-1,k}–\mu_{i-2,k+1})$$

For the full model, the predictor follows this formular:

(2) $$\mu_{\mathrm{ik}}=\mu_{11}+\left(i-1\right)\left(\mu_{21}-\mu_{11}\right)+\left(k-1\right)\left(\mu_{12}-\mu_{11}\right)+\sum_{t=3}^{i}\sum_{s=3}^{t}{∆}^{2}\alpha_{s}+\sum_{t=3}^{j}\sum_{s=3}^{t}{∆}^{2}\beta_{s}+\sum_{t=3}^{k}\sum_{s=3}^{t}{∆}^{2}\gamma_{s.}$$

The free varying parameter ξ can be defined as:

(3) $$\xi =\left(\mu 11,\mu 12,\mu 21,\Delta^{2}\alpha 3,\text{…},\Delta^{2}\alpha I,\Delta^{2}\beta 3,\text{…},\Delta^{2}\beta J,\Delta^{2}\gamma 3,\text{…},\Delta^{2}\gamma K\right)$$

Since ξ is a free varying and identified canonical parameter, different ξ value implies different predictor μ. The detailed algebraic proofs has previously been presented by Fannon. [20, 21]

Instead of working directly with age, period, and cohort, we use canonical parameter ξ to avoid the identification problem. The parameter is used to show the relationship between the dependent variable and age, period, and cohort. For example, by plotting the value of the sum of age acceleration on the y axis against age on the x axis, the relationship between age and the dependent variable can be visualized. [20] After the reparameterization, the acceleration parameters are embedded in a regression model, to test the restrictions on the model, and to include covariates in the analysis, [20] in this project, the covariates included are sex, region, and education level. All models were fitted using the apc‐package in R 4.3.3.

### 2.6 Ethical Approval

The present study was approved by the Regional Ethical Review Board of Stockholm (dnr: 2016/1001–31/4, 2020–03525; 2021–02004).

## 3 Results

### 3.1 Descriptive Analysis

Of the 2,935,147 older adults (persons older than 65 years) in the study population over the period 2006 to 2020, there were more women than men (about 55% women each year). The level of education increased over the years from 15% with the highest education in 2006 to 25% in 2020 (see Table 1).

**Table 1:** Basic characteristic of study population of older adults in Sweden, 2006–2020.

	2006	2013	2020
Sex			
Women	93722 (56%)	107618 (54.5%)	117770 (53.2%)
Men	73530 (44%)	89946 (45.5%)	103794 (46.8%)
Age groups			
65–69	44436 (26.6%)	61848 (31.3%)	55466 (25%)
70–74	35881 (21.5%)	46201 (23.4%)	57063 (25.8%)
75–79	32289 (19.3%)	33497 (17.0%)	47659 (21.5%)
80–84	27138 (16.2%)	25898 (13.1%)	30037 (13.6%)
85–89	18004 (10.8%)	18174 (9.2%)	18448 (8.3%)
90–94	7281 (4.4%)	9318 (4.7%)	9404 (4.2%)
95–99	1979 (1.2%)	2289 (1.2%)	2982 (1.3%)
100+	244 (0.1%)	339 (0.2%)	505 (0.2%)
Polypharmacy			
Yes (5 or more than 5 medications)	47754 (28.6%)	60050 (30.4%)	72718 (32.8%)
No (0–4 medications)	119498 (71.4%)	137514 (69.6%)	148846 (67.2%)

*Note:* The data used in this project includes the period from 2006 to 2020. For brevity and clarity, we selectively displayed information for the initial, median, and final years of the study period.

### 3.2 Graphic Analysis

**Figure 1:**
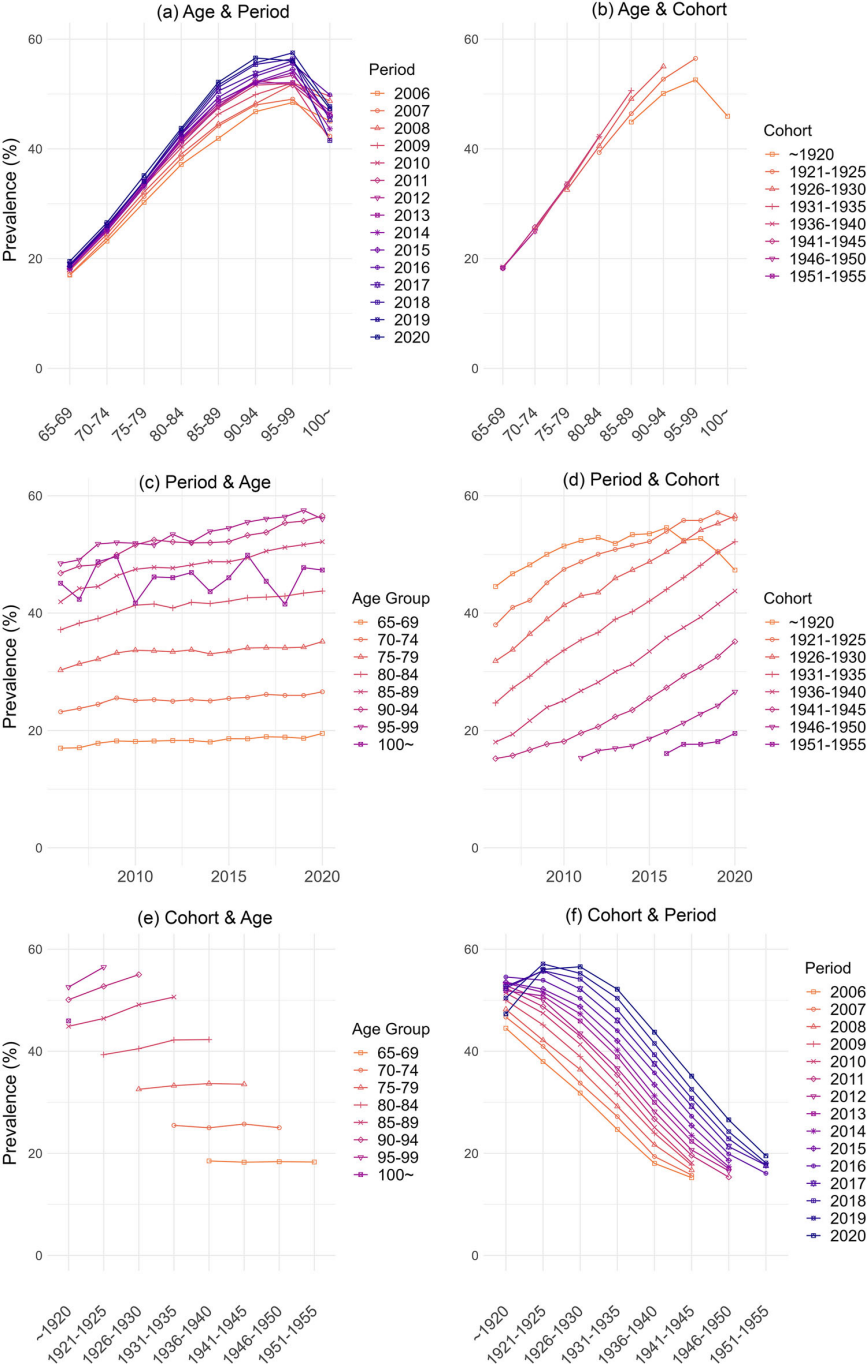
The prevalence of polypharmacy, age, period, and cohort patterns. Figure 1(a): Age trend of polypharmacy in Sweden (within periods), each line represents a period, the figure shows how polypharmacy differ across age groups within periods. Figure 1(b) Age trend of polypharmacy in Sweden (within cohort groups), each line represents a cohort group, the figure shows how polypharmacy differ across age groups within cohort groups. Figure 1(c) Period trend of polypharmacy in Sweden (within age groups), each line represents an age group, the figure shows how polypharmacy differ across periods within age groups. Figure 1(d) Period trend of polypharmacy in Sweden (within cohort groups), each line represents a cohort group, the figure shows how polypharmacy differ across periods within cohort groups. Figure 1(e) Cohort trend of polypharmacy in Sweden (within age groups), each line represents an age group, the figure shows how polypharmacy differ across cohort groups within age groups. Figure 1(f) Cohort trend of polypharmacy in Sweden (within periods), each line represents a period, the figure shows how polypharmacy differ across cohort groups within periods.

In Figure 1 we depict the age, period, and cohort effect on polypharmacy prevalence. Panel 1a illustrates the data arranged in a cross‐sectional manner. The individual lines show the prevalence of polypharmacy in each year across age groups. The prevalence of polypharmacy was consistently higher in older ages, albeit with a decline in the age groups 95–99 and 100+ years. Across the years, individuals enter the age of 65 with almost the same prevalence of polypharmacy, whereas the prevalence increases among older individuals (as indicated by the upward shift in the lines indicating more recent years (blue lines)).

Panel 1 (b) shows the data arranged by age and cohort groups. Inter‐cohort comparisons show that different cohorts have very similar prevalence among younger age groups, suggesting weak cohort effects. However, later‐born cohorts are seen to use more drugs at the same age than earlier‐born cohorts in older ages.

The period effect can be seen from Figure 1 panel (c) and (d). The parallel lines suggest weak period effects. In each period (plot c), the prevalence of polypharmacy increases modestly in all age groups except very old ones (older than 85 years old). From plot (d), every cohort have a similar slope, which suggests the effect mainly come from the age effect.

Panel (e) to (f) displays parallel lines for the majority of the plot, suggesting limited cohort effects. From plot (e), when comparing each cohort cross‐sectionally, the limited increase in polypharmacy can only be seen from people older than 85 years old, in younger age, the cohort difference is weak.

### 3.3 Subgroup Analysis

**Figure 2:**
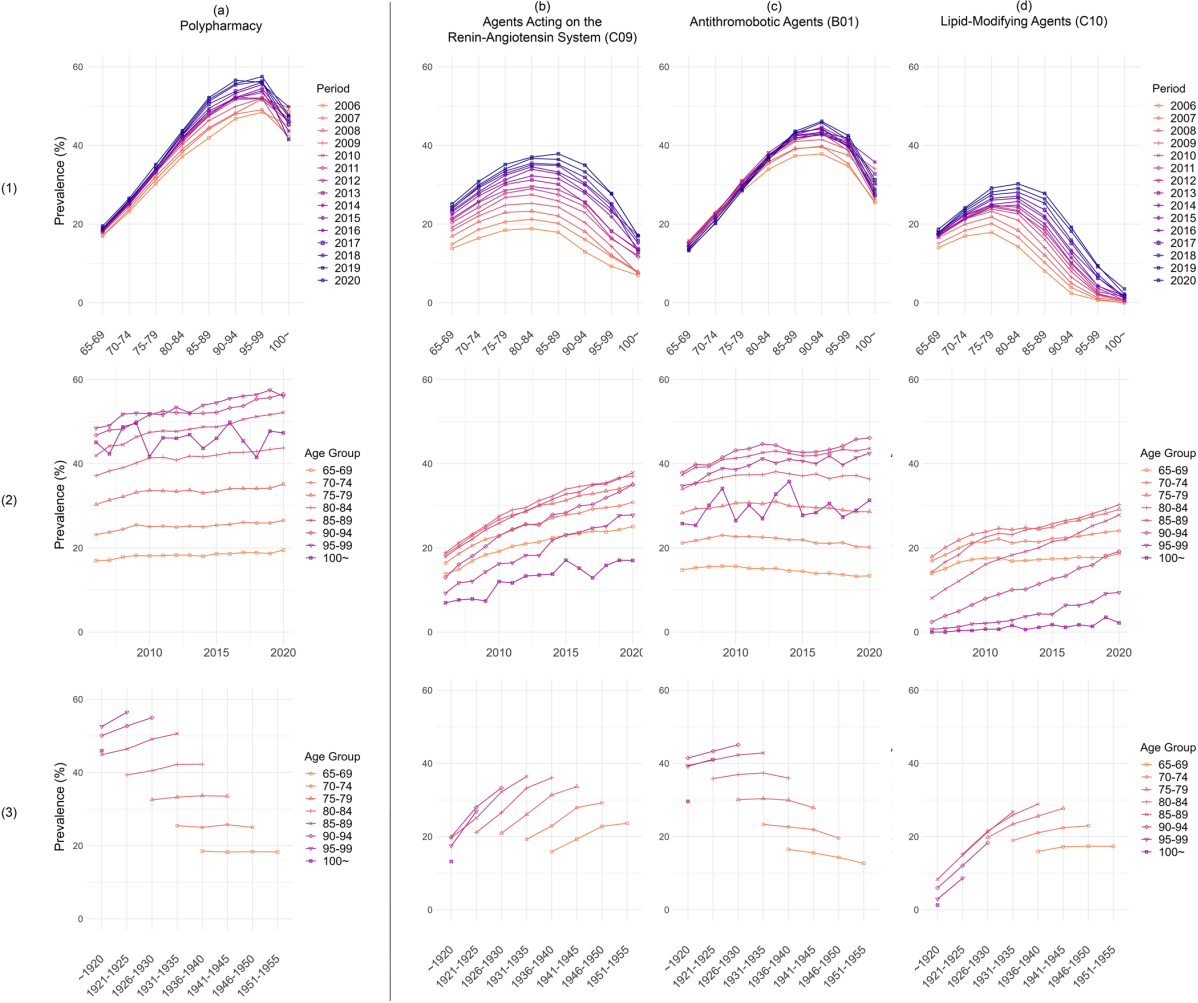
The age, period, and cohort patterns of the prevalence of polypharmacy, agents acting on the renin‐angiotensin system, antithrombotic agents, lipid‐modifying agents. Row (1): Age trend of the prevalence within periods, each line represents a period, the figure shows how the prevalence differ across age groups within periods. Row (2): Period trend of the prevalence in Sweden (within age groups), each line represents an age group, the figure shows how of the prevalence differ across periods within age groups. Row (3): Cohort trend of the prevalence in Sweden (within age groups), each line represents an age group, the figure shows how the prevalence differ across cohort groups within age groups. Column (a) plots polypharmacy. Column (b): plots agents acting on the renin‐angiotensin system. Column (c): plots antithrombotic agents. Column (d): plots lipid‐modifying agents.

In Figure 2, we compare the results for polypharmacy against the three most frequently used drug classes (agents acting on the renin‐angiotensin system, antithrombotic agents, and lipid‐modifying agents). A detailed description of each specific drug is available the supporting material, Figure S1, S2, and S3.

From panel (b) a distinct increase in the prevalence of agents acting on the renin‐angiotensin system (C09) is discernible. In plot (3b), a positive cohort effect can be observed, with the later born cohorts use more agents acting on the renin‐angiotensin system across all ages.

The prevalence of antithrombotic agents (panel c) shows a similar pattern as polypharmacy. The age effect is pronounced in the prevalence of antithrombotic agents, while plot (2c) and (3c) suggest weak period and cohort effects as indicated by horizontal lines.

The results for lipid‐modifying agents (panel d) follows a pattern like that of agents acting on the renin‐angiotensin system. The usage of lipid‐modifying agents does not exhibit an increase as at age 65 years from 2006 to 2020 (panel 1 d). Panel (2 d) displays a noticeable positive period effect in age groups older than 80 years when controlling for age, while plot (3 d) demonstrates that, within the same age group, the later‐born cohorts use more lipid‐modifying agents than those born earlier.

### 3.4 Formal APC Analysis

**Figure 3:**
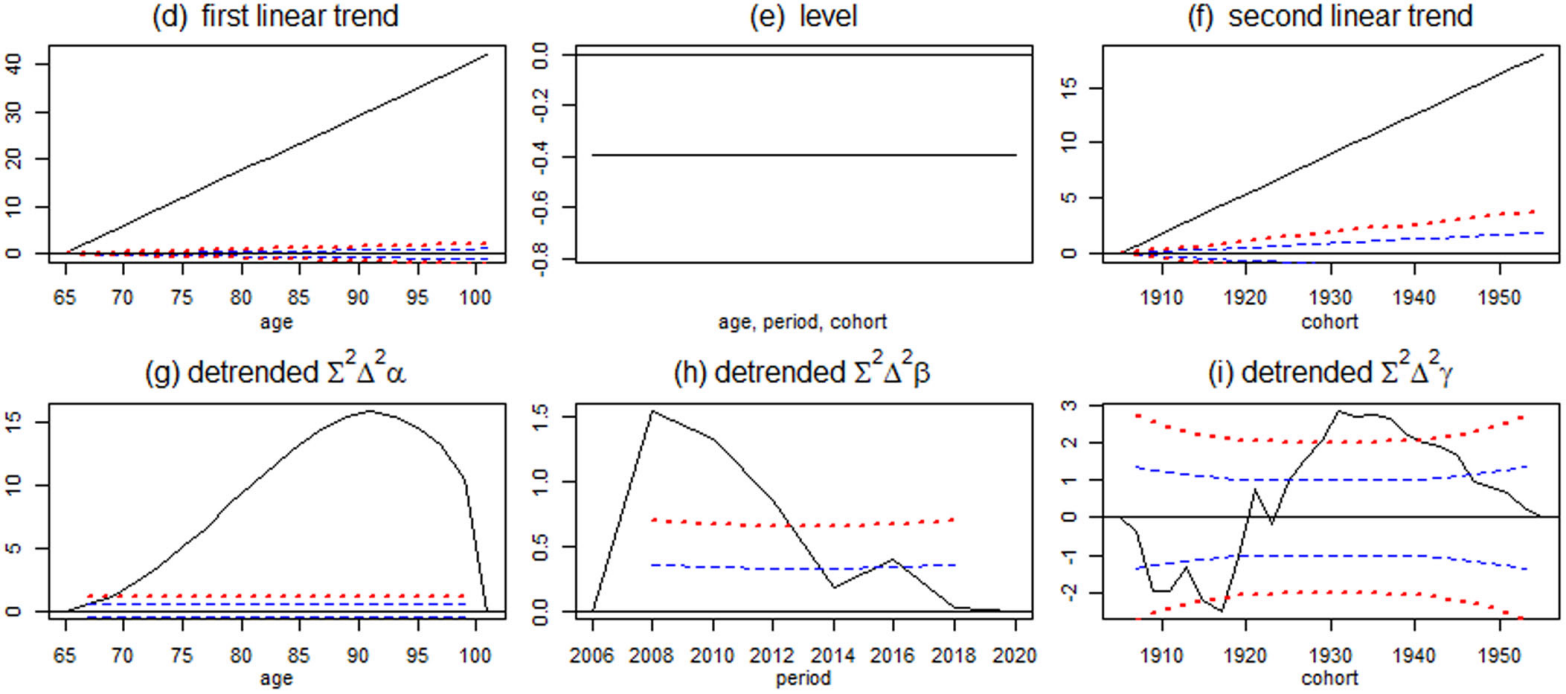
The reparametrized classical APC model on prevalence of polypharmacy. The separated linear and nonlinear development of the individual effect of age, period, and cohort. Plot (d) (e) and (f) shows the unidentifiable linear estimation of age, period, and cohort effect, and plot (g) (h) and (i) show the identifiable detrended accumulated estimated acceleration of age, period, and cohort. In the figures presented, note the varying y‐axis scales across plots. For example, a person born in 1930 was 80 years old in 2010, then the estimated prevalence of polypharmacy is the sum of the linear age trend in Figure 3 (d), the level in (e), the cohort trend in (f), the detrended age effect in (g) at age 80, period effect in year 2010 in (h) and cohort effect in 1930 in (i).

Figure 3 depicts the detrended accumulated estimated acceleration from the APC model, where the linear and nonlinear development of the individual effect of age, period, and cohort are separated. The nonlinear parts should be added above the unidentified linear trends (a more detailed algebra explanation can be seen in Nielsen [18]).

In Figure 3, a concave relationship is apparent in both age and period in plots (g) and (h), and a S‐shape in plot (i). The concave shape in age effect and period effect support that the effect from age and period are always increasing, but the acceleration drops after a certain point. In Figure 3, the age effect acceleration peaked at 90 years old (plot g), and the period acceleration peaked at 2008 (plot i). The S shape in plot (i) means the increase in cohort effect dropped until 1920s, and then started increasing. The blue and red dotted line in Figure 3 represents one and two standard deviations, if the plot line surpasses the red dotted line the effect will be considered statistically significant on a 5% significance level [18, 21]. All three plots surpass two standard deviations, this implies that age, period, and cohort nonlinearity sufficiently capture the characteristics of this data. In terms of magnitude, the age effect is the most influential, while also the detrended period and cohort accelerations are lower yet statistically significant. The age effect in both linear trend and detrended acceleration was the main effect in age, period, and cohort effect. The estimated second difference parameters can be seen in Figure S4.

The APC analysis of specific drug categories (agents acting on the renin‐angiotensin system, antithrombotic agents, and lipid‐modifying agents) also shows statistically significant age, period, and cohort effects on the prevalence (Figure S5, S6 and S7). Covariates were also analyzed in the model, the detailed results can be seen from supporting materials (Table S1).

In the sensitivity analysis, graphical analysis and APC analysis were also performed using the number of drugs as the outcome. The result look almost identical to that of the prevalence of polypharmacy.

## 4 Discussion

In this nationwide repeated cross‐sectional analysis of adults aged 65 years and older, we found that the prevalence of polypharmacy has increased from 2006 to 2020. Previous studies have mainly analyzed how age and period affects the prevalence of polypharmacy. [1] In this article, we performed an age‐period‐cohort analyses to simultaneous estimate the effect of the three time variables. The increase in polypharmacy was most pronounced in persons aged 85–89, whereas the prevalence was relatively stable at 65 years old. Cohort effects were weak for polypharmacy, but in the analysis of specific drug classes there were signs of cohort effects in the formal APC analysis.

All age groups show some increasing in polypharmacy over time, but the increase over the years have been most pronounced in 85–89 years old group, from 41.91% to 52.16% over 15 years. The increase was the highest not just in absolute percentage (10.25%), but also in relative percentage (24.4%). Strategies to reduce polypharmacy should target this age group. Interestingly, we found that people do not enter old age (defined as age 65 years) with a higher prevalence of polypharmacy. This suggest that the increasing use of preventative medications in middle age is not the main factor affecting the increasing prevalence of polypharmacy among older adults.

The positive association between age and polypharmacy have been shown repeatedly, [18] often with a J‐shape, meaning that the increase is plateauing or reverting after approximately 95‐years of age. [11] reported similar result in a Danish cohort study which contrasted cross‐sectional and longitudinal analysis. Our study does not analyze the reasons for the decreasing of drug use after 95 years old. However, it may be related to that the decrease is around the modal age of death when medical treatments tend to accumulate, the coexistence of many chronic conditions, for example, multimorbidity. Future studies should focus on the composition of drugs contributing to this increasing pace of polypharmacy.

It is well‐established that the prevalence of polypharmacy has increased during the last third decades, in line with the findings from this study. [22] We show that the increase in polypharmacy was consistent from 2006 to 2020, but faster until 2008. We found weak cohort effects for polypharmacy, whilst they were more prevalent when analyzing specific drug classes. Cohort effects have previously been found in relation to specific medications, often in relation to market entry. [23] The absence of cohort effects for polypharmacy can likely be attributed to that the composition of drugs change over time, and although some blockbuster drugs enter the market affecting specific cohorts, this effect is likely compensated by earlier or later cohorts using other medications for the same indication. We suggest that further studies on trends in general polypharmacy can focus on age and period effect, so that identification problem is avoided. But for specific drug categories, cohort effect should be further investigated.

In general, the three drug classes we analyzed displayed some differences in age, period, and cohort effects, suggesting that narrowing the scope can reveal important differences, likely in relation to different phases of the drug life cycle. Additionally, the similarity between the trend of prevalence of polypharmacy and that of antithrombotic agents could be due to the demographic shift in older population and the wide preventive usage of antithrombotic agents. [24]

### 4.1 Clinical Implications

In relation to clinical practice, our findings suggest that the increasing trend in polypharmacy is driven by a more rapid increase in polypharmacy in age group 85–89 years. It is important to understand why this increase is most pronounced at these ages, as this can inform strategies to reduce polypharmacy. The prevalence of polypharmacy showed a very modest increase at age 65 years. Thus, interventions to reduce medication use already at middle age to lower the risk of polypharmacy are potentially futile on a population level.

For the specific drug classes analyzed, agents acting on renin‐angiotensin system and lipid‐modifying agents were both impacted by cohort effects while antithrombotic agents did not show a strong cohort effect. This suggests that the cohort effect can be important for understanding the composition of drugs in polypharmacy, whereas it is of less importance for whether a person will be exposed to polypharmacy.

### 4.2 Strength and Limitation

This project's primary strength lies in being the first investigation of age, period, and cohort effects related to drug use and polypharmacy, spanning an extensive time frame from 2006 to 2020 in Sweden. To the best of our knowledge, this is the first project to comprehensively assess drug use patterns within the age‐period‐cohort framework across such a prolonged period not only in Sweden, enabling a comprehensive understanding of these effects.

Another strength of the study comes from the large and unselected study population from Swedish register data. In this project, a randomly sampled 10% of the data from the entire Swedish population between 2006 and 2020 was used. The extensive coverage reduces sample bias.

However, this project does have certain limitations. First, the study solely recorded prescribed and dispensed drugs, omitting over the counter (OTC) medications, self‐medication, and medications used in the hospitals. It was estimated that 84% of the total drug utilization was recorded in Swedish Prescribed Drug Register, [25] this could lead to potential undercoverage bias. Some commonly used OTC drugs (e.g., Aspirin) belongs to the drug category we focused on, potentially resulting in an underestimation of the selected drug category, and more generally for the polypharmacy prevalence. Second, only drug use on 1 January of each year was recorded, seasonal factors could influence the prescription and dispensation of drugs. [26] The third limitation stems from the mathematical impossibility of isolating the linear effects of age, period, and cohort, that is, the identification problem. We used canonical parameter model to avoid this problem. Fourth, it is important to note that polypharmacy does not necessarily imply inappropriate drug use. However, this project did not account for inappropriate drug use, only using the numeric count of drugs or prevalence of certain drugs as the outcome.

## 5 Conclusion

We found significant age, period, and cohort effects on drug use patterns among older adults in Sweden. We found that the prevalence of polypharmacy is continuously increasing, largely driven by a faster accumulation of medications between the ages 85–89 years over time. Cohort effects were statistically significant yet weak for general drug use, but could be identified for specific drug categories. Our results indicate that strategies aiming at reducing polypharmacy should be directed at persons 85–89 years of age with a fast accumulation of drugs, as these are the individuals contributing to the increasing prevalence of polypharmacy.

## Author Contributions

**Theodore T. Miao:** formal analysis, investigation, methodology, visualization, writing – original draft, writing – review and editing. **Malin Ericsson:** conceptualization, investigation, methodology, project administration, supervision, validation, writing – review and editing. **Jonas W. Wastesson:** conceptualization, funding acquisition, project administration, resources, supervision, validation, writing – review and editing.

## Disclosure

The authors have nothing to report.

## Consent

The present study was approved by the Regional Ethical Review Board of Stockholm (dnr: 2016/1001–31/4, 2020–03525; 2021–02004).

## Conflicts of Interest

The authors declare no conflicts of interest.

### 1 Transparency Statement

Theodore Tianyi Miao affirms that this manuscript is an honest, accurate, and transparent account of the study being reported; that no important aspects of the study have been omitted; and that any discrepancies from the study as planned (and, if relevant, registered) have been explained.

## Supporting information

Supporting materials clean TM 0416.

## Data Availability

Data from Swedish register can only be obtained by request to register‐holding authorities.
