# Supplementary material for: The Prevalence of Polypharmacy and the Contribution of Age, Period, and Cohort Effects in Sweden From 2006 to 2020
Source: Health Sci Rep. 2025 May 19;8(5):e70778. doi: 10.1002/hsr2.70778 (PMC12086637; doi:10.1002/hsr2.70778)
Supplement: Supplementary file 1 — Supporting materials clean TM 0416. [file HSR2-8-e70778-s001.docx]

**The prevalence of polypharmacy and the contribution of age, period, and cohort effects in Sweden 2006 to 2020**

**Supplementary materials**


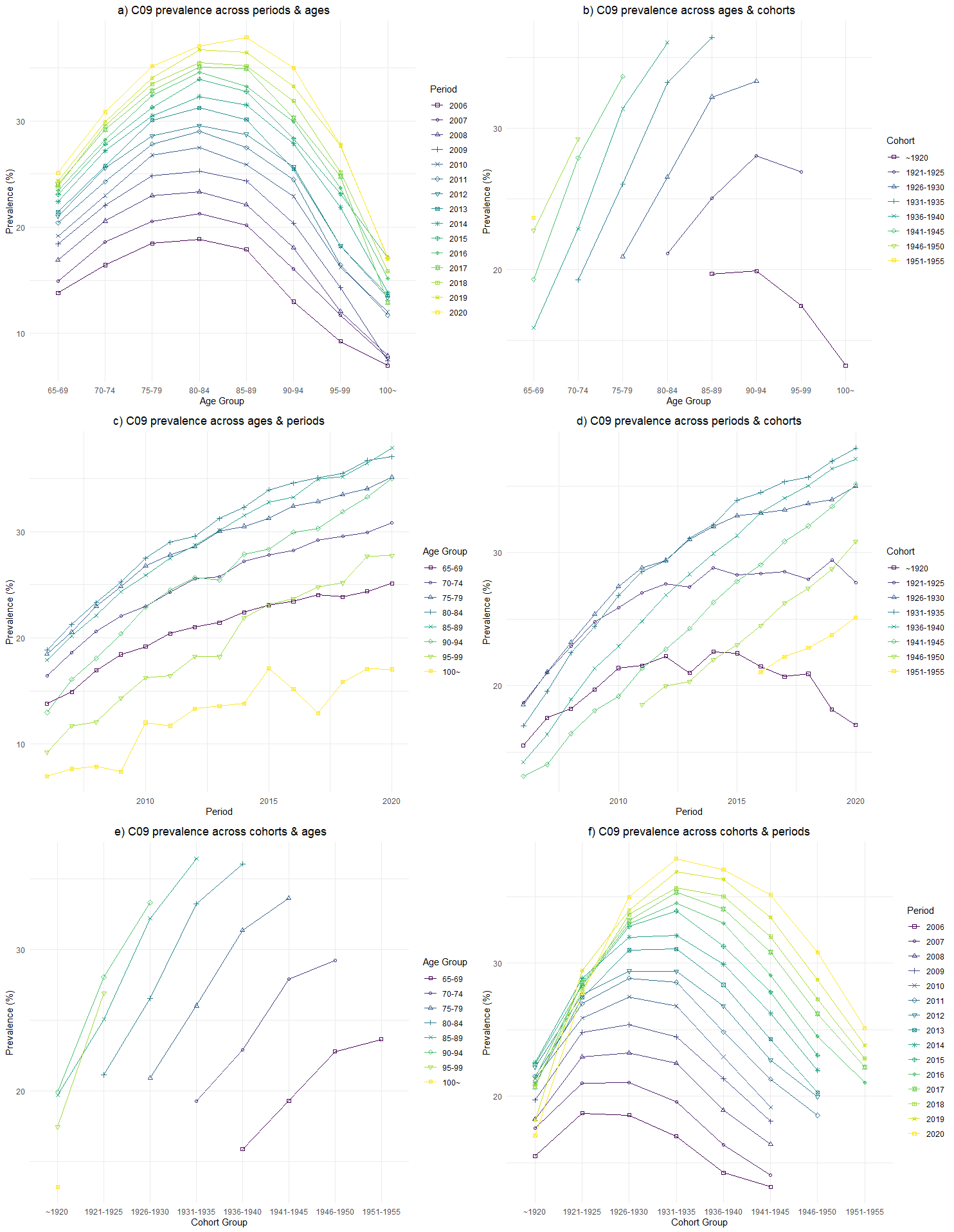


**Figure S1.** Prevalence of agents acting on the renin-angiotensin system (C09) drugs. Age, period, and cohort graphic analysis.

Figure S1 plot a) illustrates a reversed U-shaped relationship between agents acting on the renin-angiotensin system prevalence and age across different periods. The age effect is clear: the peak of agents acting on the renin-angiotensin system usage occurred within the age groups of 80 to 89 and dropped sharply, ending lower than the entry point for ages older than 100 years. In every period, after controlling for age groups, the usage of agents acting on the renin-angiotensin system is higher in each subsequent period. The density of the plots is higher in more recent periods, indicating a slower increasing rate compared to earlier periods. Notably, the entry point for agents acting on the renin-angiotensin system is significantly higher in the year 2020 than in 2006, suggesting that people are entering old age with higher agents acting on the renin-angiotensin system usage than in previous periods. A moderate period effect can be observed, particularly in the 75-94 years old age groups.

Figure S1 plot b) presents the data arranged in an age-cohort relationship. Individuals born in later cohorts enter old age with a higher prevalence of agents acting on the renin-angiotensin system usage. As the same cohort ages into the next age group (5 years older), the prevalence of agents acting on the renin-angiotensin system is 3% to 5% higher in the entire research population. An exception can be seen in the earliest-born cohort, where agents acting on the renin-angiotensin system consumption dropped dramatically after a slight increase at 90 to 94 years old. This drop can be interpreted as an age effect. It is evident that individuals enter the age of 65 with higher agents acting on the renin-angiotensin system usage in each cohort, and the highest prevalence occurs in the cohort of 1931-1935 at 85- 89 years old. The inter-cohort difference is higher in earlier-born cohorts than in later-born cohorts, indicating a stronger cohort effect among earlier-born cohorts.

The usage of agents acting on the renin-angiotensin system in different periods is depicted Figure S1 plot c). The age group of 80 to 84 has the highest prevalence of agents acting on the renin-angiotensin system usage, while the age group of 100+ has the lowest prevalence. Worth mentioning is that although the prevalence of agents acting on the renin-angiotensin system usage increases over the years within every age group, a gradual reduction in the rate of acceleration is noticeable as the upward gradient flattens out.

Figure S1 plot d) displays the period and cohort relationship. From the plot, it is evident that the 1926-1930 cohort had the highest agents acting on the renin-angiotensin system prevalence before 2012. After that, the highest prevalence is observed in the 1931-1935 cohort. Cohorts born before 1930 showed varying levels of decrease in agents acting on the renin-angiotensin system usage after 2012, while other cohorts exhibit an increasing percentage over time. Period effects can be observed in plot c) and (d) but is not strong.

Figure S1 plot e) is similar to plot b), but distributed over cohorts, illustrating the cohort and age effects. Every age group experienced a sharp increase followed by a slower increase as they move from left to right (the later-born cohorts). In general, within the same cohort, a younger age corresponds to a lower prevalence of agents acting on the renin-angiotensin system usage. Younger age groups show slower increase compared to older age groups, but the increase is still fast.

Figure S1 plot f) demonstrates cohort differences among periods, allowing for the comparison of cohort differences within the same period. In each registration year, an inverted U-shaped relationship can be observed. From the period 2006 to 2020, the peak prevalence gradually shifted from the cohort 1921-1925 to 1931-1935, after which the prevalence dropped at a steady pace. The curves exhibit a moderate period effect and cohort effect.


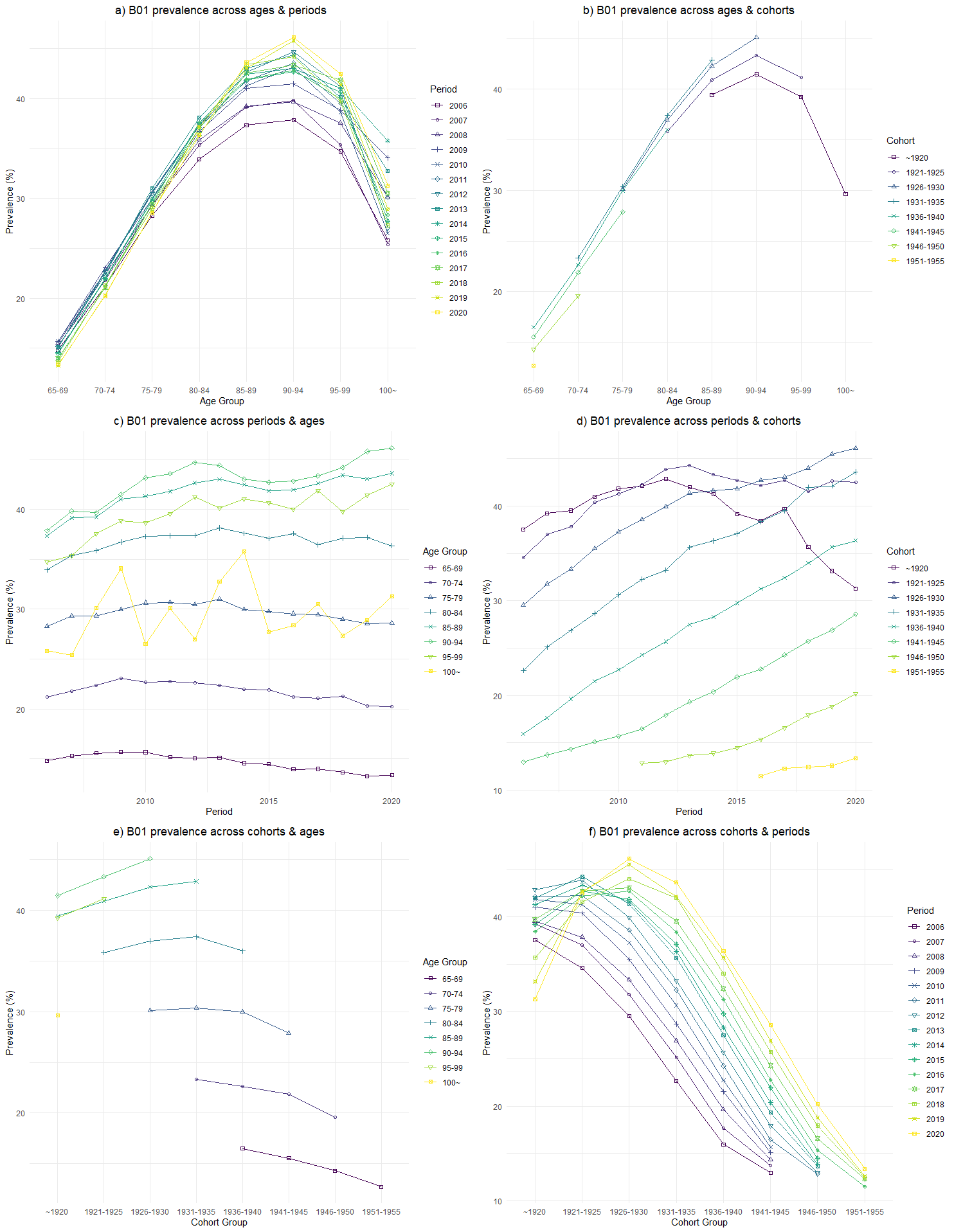


**Figure S2.** Prevalence of antithrombotic agents (B01) drugs. Age, period, and cohort graphic analysis.

Figure S2 plot a) represents the age effect controlled by periods. All the plots exhibit a similar pattern with inverted U-shaped curves, with the highest prevalence occurring at the age of 90 to 94. The increase rate is higher in the younger age group (65 to 79) than in the older age groups (80 to 94). Among different periods, individuals entering at 65 years old exhibit nearly the same prevalence of antithrombotic agents. Only very minor period differences are observable in the middle part of the curve, suggesting although the usage of antithrombotic agents is increasing every year, the period effect is not strong.

Figure S2 plot b) illustrates the age effect grouped by cohorts. In the younger age groups, the cohort curves largely overlap, but in the older age groups, slightly greater differences between the cohorts become apparent, suggesting a weak cohort effect. Later-born cohorts enter old age with a lower prevalence of antithrombotic agents drugs but surpass earlier-born cohorts later on. The cohort born between 1931 to 1935 has the highest antithrombotic agents prevalence across all cohorts when age is controlled.

Figure S2 plot c) and d) depict the period effect grouped by age and cohort, respectively. In plot (c), the period effect is barely observable among people under 84 years old, the lines representing the periods are almost parallel to the x-axis. Older groups exhibit a moderate increase in prevalence as they move from left to right.

In plot d), the period difference is still not strong when combined with cohort groups. Within a cohort, the prevalence of antithrombotic agents increases with the period, except for the earliest-born cohorts. However, this increment could be largely biased by the age effect as individuals enter older age with the increase in period.

Figure S2 plot e) demonstrate the cohort effect grouped by age. Later-born cohorts have a lower prevalence of antithrombotic agents compared to earlier-born ones. The prevalence rises as cohorts shift from left to right, with the rising trend halting at the cohort of 1931-1935, after which the prevalence gradually drops until the latest-born cohort.

Figure S2 plot f) shows the cohort effect arranged by periods. The prevalence of antithrombotic agents rises in most of the periods within the same cohort, and the increasing rate is steady according to the plot. Exceptions can be seen among the earliest-born cohorts, among which the prevalence drops as the period increases.


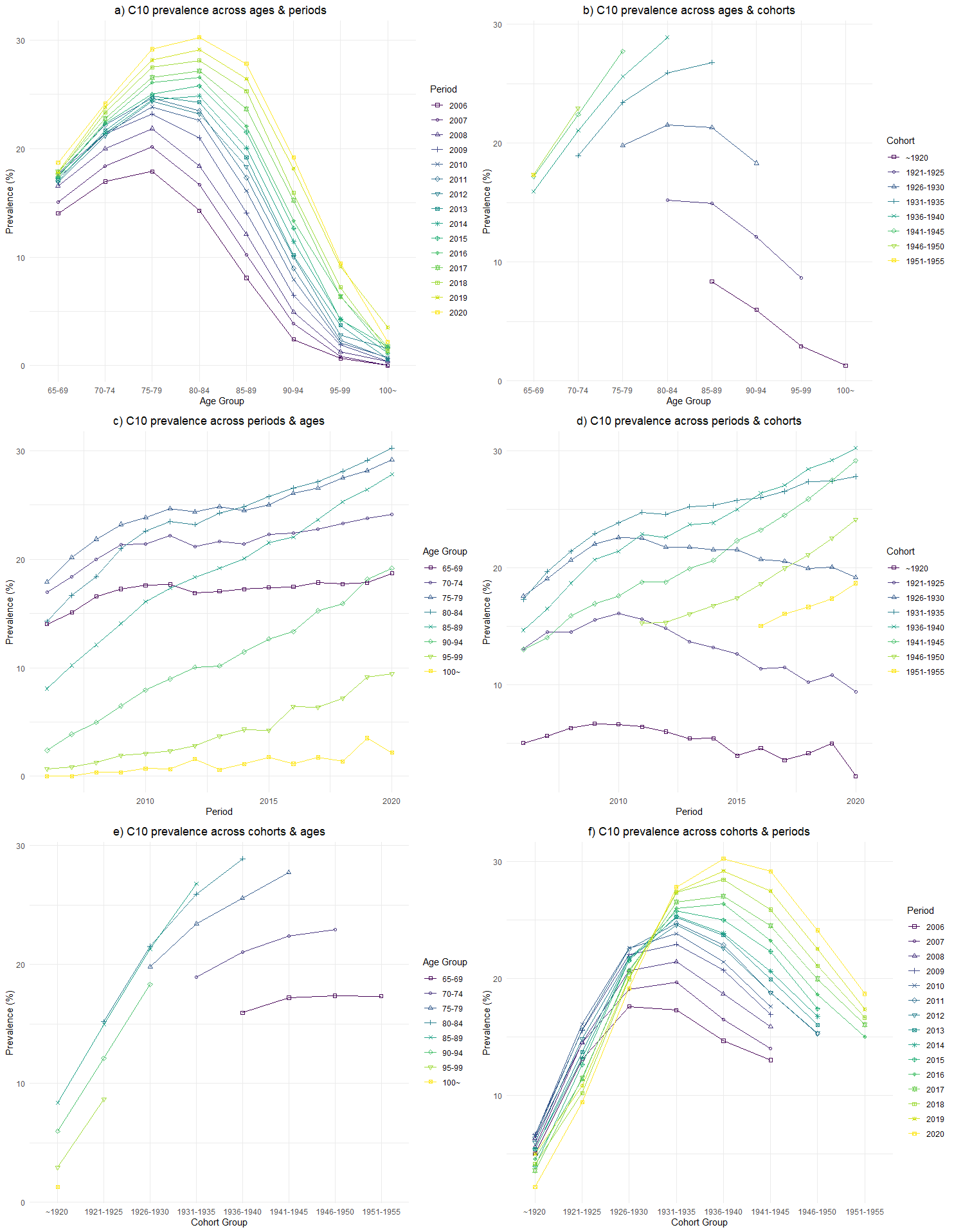


**Figure S3.** Prevalence of lipid-modifying agents (C10) drugs. Age, period, and cohort graphic analysis.

Figure S3 plot a) displays the age-period relationship. It can be observed from plot a) that, although the difference is not substantial, in more recent periods, people enter old age with a higher prevalence of lipid-modifying agents. For periods before 2013, the curves peak in the 75-79 years old age group. Conversely, for more recent periods, the curves consistently rise until they peak in the 80-84 years age group. Following the peak prevalence, the usage drops rapidly, and all curves converge in the oldest age groups, showing a similar prevalence regardless of the period. A similar pattern can be observed in plot b), which illustrates the age and cohort difference. People enter old age with a slightly higher prevalence in recent cohorts, and the usage peaks at the age of 75-84 years old before dropping rapidly after the peak. The inter-cohort difference is larger in the earlier-born cohorts, suggesting stronger cohort effects when controlling for age. According to the first-order relationship in plot b), later-born cohorts enter old age with a slightly higher prevalence in C10.

Figure S3 plot c) and d) show the period effect grouped by age and cohort respectively. In most age groups, the prevalence increases over periods, though the increase is small in the younger age groups. The earlier-born cohorts (before 1930) experienced only a slight rise in prevalence, followed by a slow drop after peaking in 2010. Other cohort groups continued to rise over the years. A noisy period effect can be observed in plot c) and d) when combined with age and cohort effects.

Figure S3 plot e) and (f) depict the cohort differences by age and period. When controlling for age groups, the later-born cohorts exhibit higher prevalence at various levels. The fastest increment is seen in the earlier-born cohorts, which slows down after the 1930s. The later- born cohorts not only had a lower acceleration but also a lower overall prevalence of lipid-modifying agents. This could be due to a lower need for lipid-modifying agents in younger ages or cohort differences. However, it is reasonable to hypothesize a mutual effect from age and cohort. From plot f), the cross- sectional differences can be observed, and the overlapping plots suggest low period effects. Cohorts born after the 1930s show a higher inter-period difference. Taken together, although the cohort and period effects are not as significant as the age effect compared to plot a) and b), they still play a role in the change of lipid-modifying agents prevalence.

**
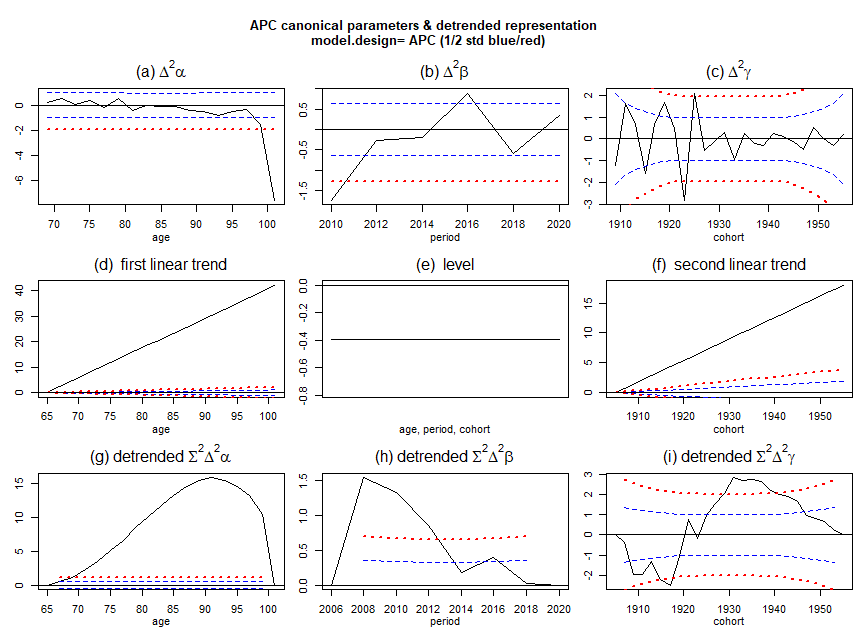
**

**Figure S4.** Time effects, APC model of prevalence of polypharmacy.

The top row of Figure S4 shows the estimated acceleration from the APC model, the detrended accumulated estimated acceleration is shown on the bottom row (Plot g to i).

The middle row (Plot d to f) does not have natural interpretation because of unidentified period linear effect is also combined into plot (d) and (f), their function is to create the third row of Figure S4, to make sure the detrended acceleration plots anchored to zero at the start and end point. However, because of the identification problem of APC analysis mentioned above, the linear trend in age, period, and cohort effects are not fully identifiable, thus, plot (d) to (f) cannot be seen as the most precise line of solution. Although they do not have natural interpretation, can still give us a hint of the increasing trend of drug use with increasing age and cohort, consequently, the drug use should also increase with period ^20^. The trend from plots (g) to (i) should be added to the linear trend presented in plots (d) to (f) to create an actual line of solution. However, because of the nature of plot (d) to (f), it is more precise to only look at the non-linear part of the curves (the bottom row of Figure S4). Within Figure S4, a concave relationship is apparent in both age and period in plots (g) and (h), and a S shape in plot (i). The concave shape in age effect and period effect means the effect come from the age and period are always increasing, but the acceleration drops after a certain point. In Figure S4, the age effect acceleration peaked at 90 years old (plot g), and the period acceleration peaked at 2008 (plot i). The S shape in plot (i) means the increase in cohort effect dropped less and less until 1920s, and then started increasing. In terms of magnitude, while the distinct detrended period and cohort accelerations are significant, the age effect remains the most influential (note the scales in y axis). The age effect in both linear trend and detrended acceleration was the main effect in the three.

**
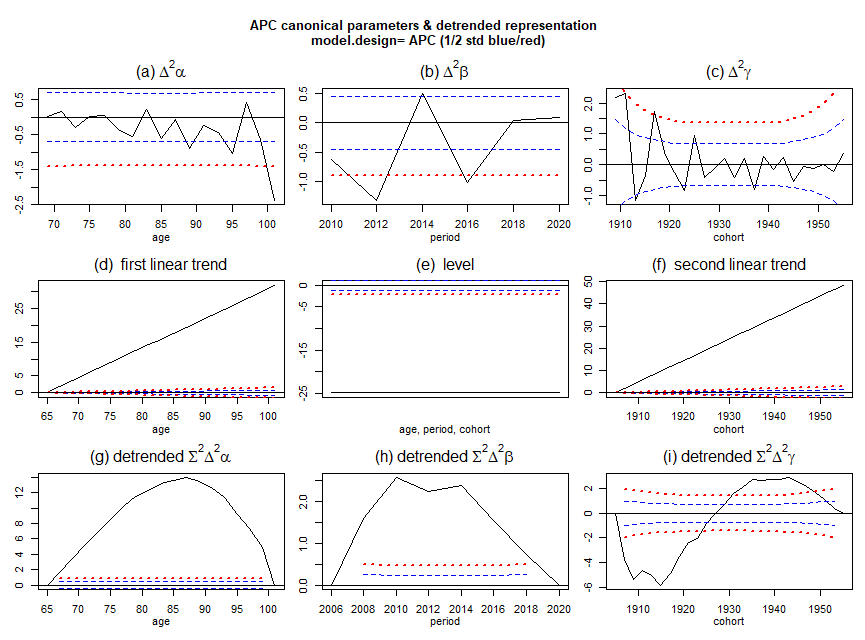
**

**Figure S5.** Time effects, APC model of prevalence of agents acting on the renin-angiotensin system (C09)

Starting with the middle row of Figure S5, the increasing trend of C09 usage in age, period, and cohort effect can be observed. Note the increasing trend can be only seen as an general interception.

Plot (g) and (h) exhibit a smooth concave shape in the detrended acceleration, except for the exception seen in the drop at the year 2010, which is the only year causing a non-constant pattern. Which lead to the conclusion of the acceleration in age increased until 85 to 90 years old, and the acceleration in period was peaked in 2014. As for plot (i), it displays convexity up until the early 1920s and transitions to concavity thereafter, indicates that the increase from cohort effect dropped less and less until late 1920s, and then started increasing.

In terms of magnitude, the effect from age was the strongest, while the three time effects were all significant.

**
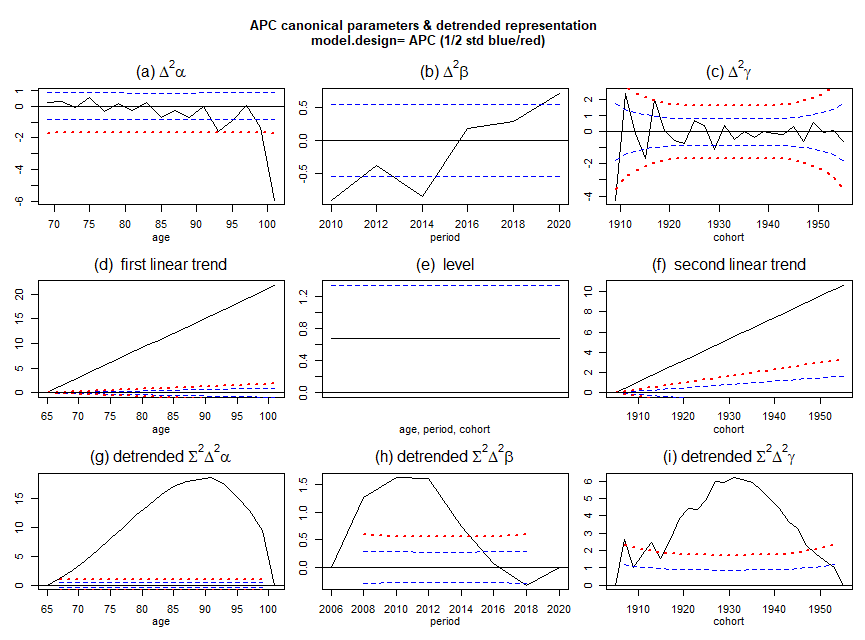
**

**Figure S6.** Time effects, APC model of prevalence of antithrombotic agents (B01) prevalence

Plots (d) and (f) illustrate the general increasing usage trend of antithrombotic agents, corresponding to increasing age, period, and cohort. In plots (a) to (c), we observe that the acceleration of age and cohort exhibits a wavy pattern, with a significant drop in acceleration occurring in older age groups and earlier-born cohorts. This drop can be attributed to the data’s sparsity.

Plots (g) to (i) display a smooth concave shape across all three time effects. The increase in age, period, and cohort all demonstrates a near-quadratic relationship with the detrended acceleration. In another way, the increase in prevalence of antithrombotic agents from age effect growed faster and faster until 90 years old, and then the increase slowed down. For period effect, the acceleration was fastest in 2010 to 2012, and slowed down until that, in the year of 2016, the acceleration started to decrease. For cohort effect, the acceleration was fastest until 1930s, and then dropped after that. As is consistent with all other models, the detrended age effect remains dominant in the three time effects (note the different scale in y axis). While period and cohort effects are also significant, their non-linear contributions are smaller compared to the age effect.

In terms of magnitude, the effect from age was the strongest, the effect from cohort was the second strong, while period effect is weak.

**
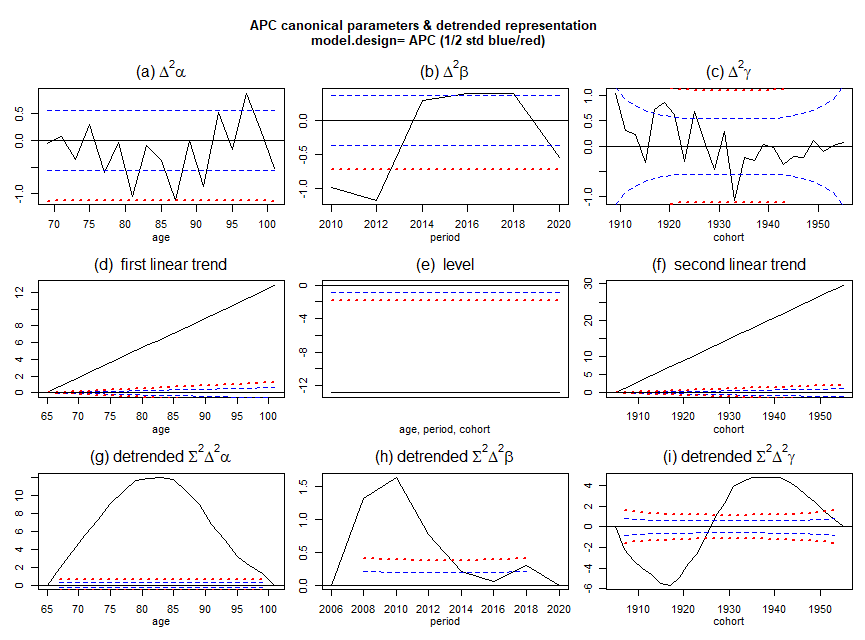
**

**Figure S7.** Time effects, APC model of prevalence of lipid-modifying agents (C10)

Plots (d) to (f) depict an increasing trend in time effects and lipid-modifying agents usage. Referencing Figure S7 (b), a significant acceleration in the prevalence of lipid-modifying agents is evident in the year 2014. Looking at the first row, there isn’t a clear pattern of age and cohort acceleration. Nevertheless, when examining the accumulated acceleration plots (the bottom row of Figure S7), a concave shape in age and period becomes noticeable. The increase in prevalence of lipid-modifying agents reached a peak at 80-85 years old in age dimention and in the year of 2010 in period dimention. A S-shaped curve can be seen from the detrended accumulated cohort acceleration in Figure S7 (i). The increase of prevalence of lipid-modifying agents in cohort effect dropped less and less until 1925 and then started increasing after that. In terms of magnitude, the effect from age was the strongest, and then, the cohort effect. While the three time effects were all significant, period effect was the weakest.

**Table S1. Covariates**

|  | Number of drugs | Polypharmacy | Agents acting on the renin-angiotensin system (C09) | Antithrombotic agents (B01) | Lipid-modifying agents (C10) |
| --- | --- | --- | --- | --- | --- |
| *Sex* |  |  |  |  |  |
| Women | *ref.* | *ref.* | *ref.* | *ref.* | *ref.* |
| Men | -0.256*** | -0.097*** | 0.251*** | 0.499*** | 0.356*** |
| *Education* |  |  |  |  |  |
| Pre-secondary | *ref.* | *ref.* | *ref.* | *ref.* | *ref.* |
| High school | -0.157*** | -0.094*** | -0.055*** | -0.053*** | -0.059*** |
| University and higher | -0.546*** | -0.350*** | -0.251*** | -0.249*** | -0.279*** |

**County is adjusted for when conducting regression analysis. “***” means p-value < 0.01.**

There are sex differences and education differences in drug use in pattern in Sweden (Table S1). In general, women have 9.7% higher chance of exposing to polypharmacy, and use 0.256 more drugs in number than men, while men have 25.1% higher prevalence in agents acting on the renin-angiotensin system, 49.9% higher prevalence of antithrombotic agents, and 35.6% higher prevalence of lipid-modifying agents.

For education level, people with higher education level use statistically significantly less drugs. Comparing to people with only pre-secondary level of education, people with university and higher education use 0.546 less of drugs in number, and have 35% less chance of exposed to polypharmacy. For three types of drug categories, the similar difference was also statistically significant.
